# Acupoint catgut embedding: a potential intervention strategy for obesity-related precocious puberty

**DOI:** 10.3389/fendo.2024.1448111

**Published:** 2024-10-14

**Authors:** Yun Liang, Yuan Yuan, Jie Yang

**Affiliations:** ^1^ Acupuncture and Tuina School, Chengdu University of Traditional Chinese Medicine, Chengdu, Sichuan, China; ^2^ Department of Pediatric Surgery, Affiliated Hospital, North Sichuan Medical College, Nanchong, Sichuan, China

**Keywords:** acupoint catgut embedding, precocious puberty, obesity, vaginal opening, luteinizing hormone, follicle-stimulating hormone, estradiol

## Abstract

**Introduction:**

Obesity-related precocious puberty is induced by obesity, and acupoint catgut embedding (ACE) therapy is known to treat obesity. This study aims to validate the hypothesis that ACE can delay the onset of obesity-related precocious puberty.

**Methods:**

Female Sprague-Dawley rats, 21 days old, were randomly divided into three groups: the high-fat diet combined with ACE treatment group (ACE), the high-fat diet group (HFD), and the normal control diet group (NCD), with 8 rats in each group. The vaginal opening (VO) time was monitored, and serum levels of luteinizing hormone (LH), follicle-stimulating hormone (FSH), and total estradiol (E2) were measured, followed by statistical analysis.

**Results:**

Kaplan-Meier survival curves, with VO as the endpoint, showed that vaginal opening was delayed in the ACE group compared to the HFD group, with a statistically significant difference (p < 0.05). The changes in levels of FSH, LH, and E2 indicated that sexual development was delayed in the ACE group compared to the HFD group and was more similar to the NCD group.

**Discussion:**

Combining the vaginal opening time and changes in hormone levels, this study confirms the potential role of ACE in delaying the onset of obesity-related precocious puberty.

## 1 Introduction

Precocious puberty, characterized by the premature onset of puberty prior to the age of 8 in girls and 9 in boys, is a condition occurring approximately 2-2.5 standard deviations ahead of the normative age (1). Precocious puberty can lead to a range of psychological, physiological, and social adaptation challenges for children (2). On the psychological front, children experiencing early puberty may grapple with feelings of inadequacy, anxiety, and social integration difficulties. Physiologically, this condition might result in the premature fusion of growth plates, impacting their ultimate stature. Regarding social adaptation, children with precocious puberty may find themselves alienated among their peers, facing challenges in assimilation. The global prevalence of precocious puberty is increasing, with a particularly notable uptick in cases reported during the COVID-19 pandemic in countries such as Germany and Argentina (3). In China, the rate of precocious puberty among children is 0.43%, with girls being affected 5 to 10 times more frequently than boys (4).

Obesity-related precocious puberty in childhood, which is a form of precocious puberty, is closely linked to body weight and hormonal levels (5). Studies have revealed that the adipose tissue in obese children can produce estrogen, potentially accelerating the maturation of the gonads and precipitating precocious puberty (6). Furthermore, obesity may exacerbate the condition by affecting insulin and leptin levels, thereby promoting the development of precocious puberty (7). The therapeutic approaches to Obesity-related precocious puberty are multifaceted, encompassing pharmacological treatments, behavioral interventions, and modifications to nutrition and lifestyle practices. Pharmacotherapy often employs hormone-based drugs, such as gonadotropin-releasing hormone analogs (GnRHa), to curb the premature development of the gonads (8). Behavioral interventions aim to foster the development of wholesome lifestyle habits and social competencies in children. Nutritional and lifestyle adjustments advocate for a judicious diet and consistent engagement in physical activities (9). In general, weight reduction stands as the principal intervention to mitigate the onset of obesity-related precocious puberty.

Acupoint catgut embedding (ACE) is a therapeutic technique that combines Chinese acupuncture with modern medical practices. The strategic implantation of absorbable sutures into designated acupoints activates the autonomic nervous system, increases energy expenditure, and promotes fat metabolism, ultimately facilitating weight reduction (10).

Based on the logical connection that obesity is the root cause of obesity-related precocious puberty and considering the capacity of ACE to treat obesity, the author of this paper proposes a theoretical hypothesis that ACE may have the potential to delay the onset of precocious puberty associated with obesity. To substantiate this theoretical hypothesis, the current study was conducted.

## 2 Materials and methods

### 2.1 Animal selection and grouping

Selected female Sprague-Dawley (SD) rats of specific pathogen-free (SPF) grade, weaned at 21 days post-partum with an average body weight of 45 ± 5g, were randomly assigned to three groups, each consisting of eight individuals (11). The experimental group underwent acupoint catgut embedding (ACE) and was subjected to a high-fat diet (Diet D12451, 45% fat content, supplied by Nantong Trophic Feed Technology Co., Ltd.) to induce obesity. The model control group consumed the high-fat diet (HFD), whereas the baseline control group was fed a normal chow diet (NCD) (Diet LAD 2002, 10% fat content, also supplied by Nantong Trophic Feed Technology Co., Ltd.). The rats were housed individually in a regulated environment with a temperature of 24 ± 2°C, relative humidity of 50% ± 10%, and an illuminance of 25 lux under a 12-hour light-dark cycle, with unrestricted access to food and water. The experimental group received suture embedding at the ST44 and ST36 acupoints (utilizing 4-0 polydioxanone sutures), beginning on the day of their inclusion and performed every 7 days thereafter, alternating between the left and right sides.

### 2.2 Measurement indicators

**Figure 1:**
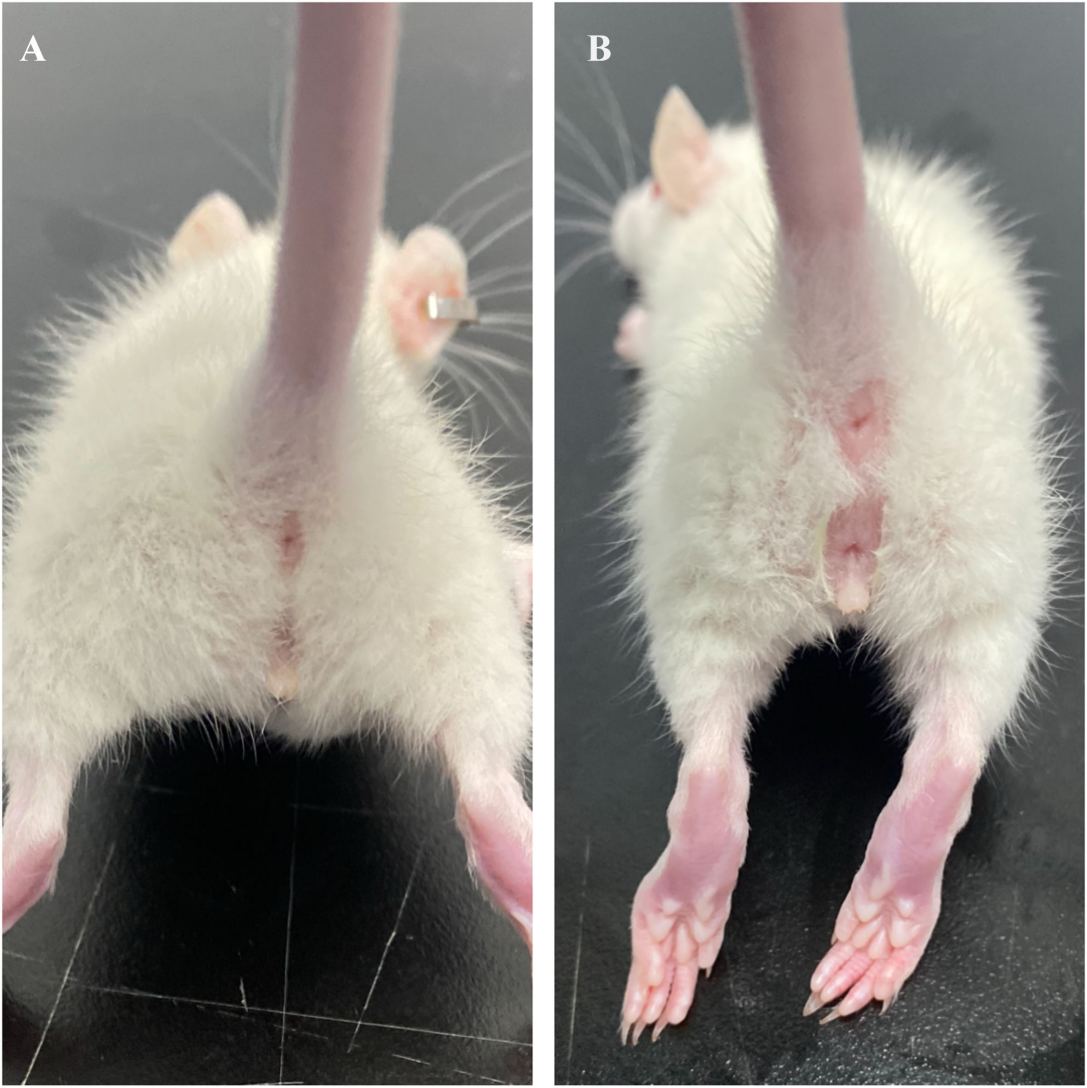
Visual representation of vaginal opening in SD rats. **(A)** Unopened; **(B)** Opened.

In female mice, the appearance of the vaginal opening (VO) signifies sexual maturity, as depicted in **Figure 1**. Observers meticulously record the onset of sexual maturation by examining the vaginal opening in rats at approximately 8 a.m. daily. This specific time is selected to align with the circadian rhythm of the female mice, ensuring uniformity in observations. Sexual maturity is deemed to have been achieved in both the ACE group and the HFD group upon the manifestation of vaginal openings, marking this moment as the conclusion of the experiment. At the inception and culmination of the study, blood samples are extracted from each rat for analysis. The serum levels of luteinizing hormone (LH), follicle-stimulating hormone (FSH), and total estradiol (E2) are quantified through radioimmunoassay, a technique renowned for its high sensitivity and specificity, thus precisely capturing the fluctuations in sex hormone levels.

### 2.3 Statistical analysis

Data were statistically analyzed and plotted using GraphPad Prism 9.0 software (GraphPad Software Inc., USA). Student’s t-test was applied for the comparison of two paired samples. For multiple group comparisons, one-way analysis of variance (ANOVA) was utilized, followed by Duncan’s or Turkey’s multiple comparison tests. A statistically significant difference was indicated by P < 0.05.

## 3 Results

### 3.1 The timing of vaginal opening

**Figure 2:**
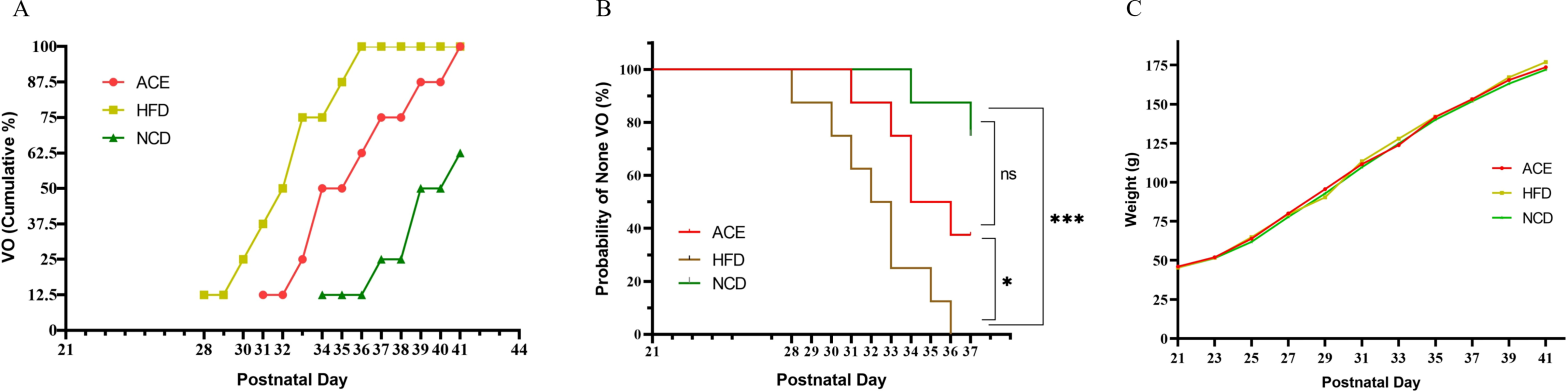
The relationship between vaginal opening and body weight gain with postpartum age in Sprague-Dawley rats. **(A)** Time distribution of vaginal opening; **(B)** Survival curves with vaginal opening as the outcome measure; **(C)** Temporal trends in body weight gain. ns indicates no statistical significance; *** denotes p<0.001, and * signifies p<0.05.

**Figure 2A** delineates that the HFD group initially exhibited vaginal opening on the 28th postpartum day, with the latest instance occurring on the 36th postpartum day. The first observation of vaginal opening in the ACE group was on the 31st postpartum day, culminating on the 41st postpartum day, which was designated as the experimental endpoint. Within the NCD group, vaginal opening was initially noted on the 28th postpartum day, and by the endpoint, three rats had yet to display this characteristic. **Figure 2B** presents the Kaplan-Meier survival curves for the three groups, with vaginal opening as the metric for outcome. As shown in **Figure 2B**, no statistically significant divergence is observed between the ACE and NCD groups; however, notable differences emerge when comparing the ACE to the HFD groups and the HFD to the NCD groups.

### 3.2 Rat body weight

On postpartum day 21, the commencement of group allocation, the body weights of the three SD rat cohorts were meticulously documented. The ACE group boasted an average weight of 46.0 ± 2.2g, the HFD group weighed in at 45.3 ± 2.6g, and the NCD group registered 45.3 ± 2.8g. At this juncture, no statistically significant discrepancies were noted among the groups (P>0.05). However, by postpartum day 23, the HFD and NCD groups began to demonstrate statistically significant variations in body weight, in contrast to the ACE and NCD groups which displayed no such notable divergence, and a pronounced difference emerged between the ACE and HFD groups.

**Figure 2A** illustrates that during the interval from postpartum days 25 to 29, the body weight trajectories of the ACE and HFD groups commenced convergence. Upon reaching postpartum day 29, the ACE group was subjected to a second round of acupoint catgut embedding. **Figure 2C** reveals that the weight gain curves of the three groups commenced divergence, signifying statistical differences among them. Upon the study’s culmination on postpartum day 41, the HFD group reached a body weight of 189.4 ± 2.8g, exhibiting a 10.05% increase relative to the NCD group’s weight of 172.1 ± 2.7g; the ACE group’s weight of 182.4 ± 2.2g was elevated by 5.98% in comparison to the NCD group.

### 3.3 Rats’ levels of LH, FSH, and estradiol E2

**Figure 3:**
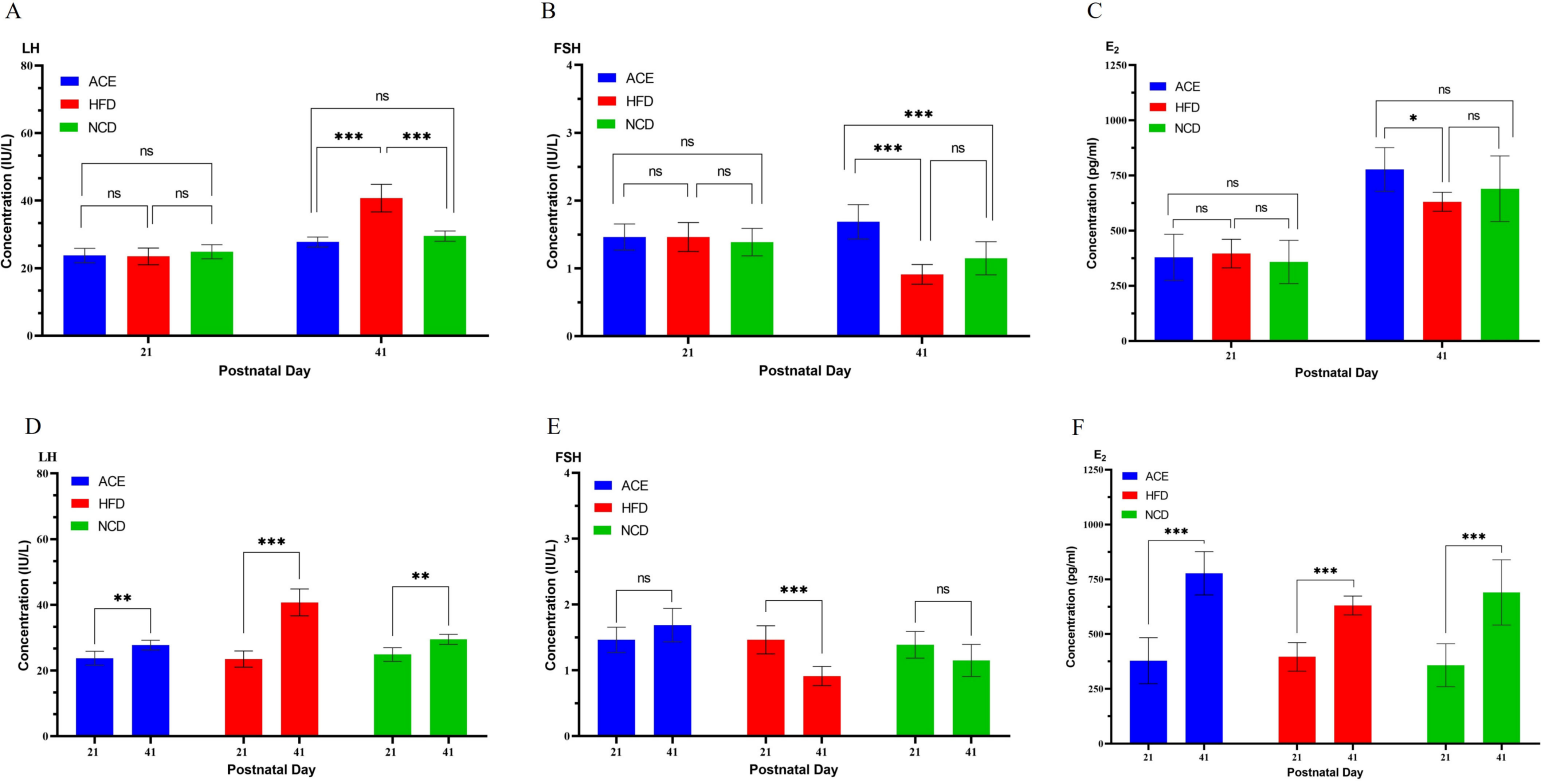
Serum levels of LH, FSH, and E2 in Sprague-Dawley rats at the commencement and conclusion of the experiment. **(A, D)** LH; **(B, E)** FSH; **(C, F)** E2. ns indicates no statistical significance; *** denotes p<0.001, ** represents p<0.01, and * signifies p<0.05.

**Figure 3A** presents the initial LH levels among the three rat cohorts on postpartum day 21, with the ACE group at 23.75 ± 2.12 IU/L, the HFD group at 23.50 ± 2.45 IU/L, and the NCD group at 24.88 ± 2.10 IU/L, revealing no significant statistical divergence. By the study’s conclusion on postpartum day 41, a marked escalation in LH levels was observed, with ACE group at 27.75 ± 1.49 IU/L, HFD group at 40.75 ± 4.10 IU/L, and NCD group at 29.50 ± 1.51 IU/L, highlighting significant statistical variations between the ACE and HFD groups and between the HFD and NCD groups, yet no discernible difference between the ACE and NCD groups.

**Figure 3B** illustrates the FSH profiles, with day 21 measurements of 1.46 ± 0.19 IU/L for ACE group, 1.46 ± 0.21 IU/L for HFD group, and 1.39 ± 0.20 IU/L for NCD group, indicating no significant statistical differences. By day 41, FSH levels shifted to 1.69 ± 0.25 IU/L for ACE group, 0.91 ± 0.15 IU/L for HFD group, and 1.15 ± 0.25 IU/L for NCD group, with pronounced statistical distinctions noted between ACE and HFD groups, and ACE and NCD groups, but not between HFD and NCD groups.

**Figure 3C** gracefully portrays the progression of E2 levels, commencing at postpartum day 21 with levels of 378.75 ± 104.94 pg/ml for ACE, 396.13 ± 64.89 pg/ml for HFD group, and 358.25 ± 98.02 pg/ml for NCD group, without significant statistical variation. Advancing to the final evaluation on postpartum day 41, a pronounced upsurge in E2 levels was noted, reaching 777.50 ± 98.71 pg/ml for ACE group, 630.63 ± 43.11 pg/ml for HFD group, and 690.13 ± 148.89 pg/ml for NCD group, with a significant statistical divergence between ACE and HFD, while no significant differences were observed between ACE and NCD groups, or HFD and NCD groups.

**Figures 3D–F** show the longitudinal comparison of hormone levels in the ACE, HFD, and NCD groups on day 41 postpartum compared to day 21. Comparing the hormone levels of SD rats on day 21 postpartum (before vaginal opening) to those on day 41 postpartum (after vaginal opening), the changes in the ACE group were: increased LH, increased E2, and no change in FSH; the changes in the HFD group were: increased LH, increased E2, and decreased FSH; the changes in the NCD group were: increased LH, increased E2, and no change in FSH.

## 4 Discussion

The key endocrine system responsible for regulating sexual development is the Hypothalamic-Pituitary-Gonadal Axis (HPG Axis) (12). The HPG axis involves multilevel regulation of the hypothalamus, pituitary gland, and gonads. Under normal conditions, the hypothalamus periodically secretes gonadotropin-releasing hormone (GnRH) (13). GnRH reaches the anterior pituitary through the hypothalamic-pituitary portal system, stimulating the secretion of LH and FSH (14). The secreted LH and FSH circulate through the bloodstream to the gonads (ovaries or testes), promoting their development and the synthesis of sex hormones (estrogen, progesterone, and testosterone) (14). The HPG axis maintains sex hormone balance and regulates puberty onset, menstrual cycle, and reproductive function through feedback mechanisms involving GnRH, LH, FSH, and sex hormones (14). In the general population, the activation of the HPG axis follows a relatively fixed schedule, primarily determined by genetic factors and physiological development (15) Girls typically begin between ages 8 and 13, while boys start between ages 9 and 14 (16).

Obesity-related precocious puberty refers to the clinical phenomenon where sexual development is advanced due to obesity. The primary physical characteristic of these patients is a significantly higher amount of body fat and body mass index compared to the normal range for children of the same age. Adipose tissue not only stores energy but also has endocrine functions, secreting various hormones and cytokines (e.g., leptin, adiponectin) (17). Leptin, which is secreted by adipocytes (especially white adipose tissue), maintains energy balance by regulating appetite and metabolism (18). The amount of leptin secreted is directly related to the total amount of adipose tissue; thus, obese children have significantly elevated leptin levels due to the increased number and size of adipocytes (18). The HPG axis in obese individuals is activated prematurely due to high leptin levels (19). Elevated leptin levels reach the hypothalamus through the bloodstream, acting on leptin receptors in the arcuate nucleus to increase the pulsatile release of GnRH (19). Additionally, obese children are often accompanied by insulin resistance (20). Insulin resistance leads to elevated insulin levels, resulting in increased secretion of insulin-like growth factor-1 (IGF-1) (21), decreased production of sex hormone-binding globulin (SHBG) (22), and increased levels of free sex hormones (23). The combined effects of insulin resistance and high leptin levels in obese children lead to the premature activation of the HPG axis, disrupting its feedback mechanisms and ultimately resulting in precocious puberty (24).

Weight loss can reduce adipose tissue, particularly visceral fat, improving insulin sensitivity in the liver and muscles, thereby lowering hyperinsulinemia. Additionally, reducing adipose tissue can also lower leptin levels. Therefore, weight loss is the primary intervention for reducing obesity-related precocious puberty (25). ACE for weight loss is a method developed based on traditional Chinese medicine theory, involving the insertion of absorbable protein threads (e.g., catgut or other medical absorbable threads) into specific acupoints to stimulate them, promoting metabolism and increasing energy expenditure, thereby achieving weight loss (26).

This paper proposes the hypothesis that ACE may delay the onset of obesity-related precocious puberty. To test this hypothesis, we conducted an animal study.

### 4.1 Selection of experimental animals

A systematic review conducted by Song Yongfu and colleagues examining the correlation between precocious puberty and the risk of obesity in children has indicated a significant association between early puberty in girls and an elevated risk of obesity (5). In contrast, a meta-analysis of precocious puberty in boys did not reveal a connection with an increased risk of obesity ^5^. Therefore, precocious puberty in girls should be recognized as an independent risk factor for obesity. Furthermore, the sexual maturation of female SD rats is stimulated by the hypothalamus through pulsatile secretion of gonadotropin-releasing hormone (GnRH), a mechanism that mirrors the human hypothalamic-pituitary-gonadal (HPGA) axis (27). Additionally, SD rats are sensitive to sex hormones (28), which justifies the choice of female rats as the experimental model in this study.

### 4.2 Establishment of a precocious puberty model

Various methods exist for constructing an animal model of precocious puberty, including induction via danazol (29), melatonin (30), and E_2_ (31), as well as the high-fat diet (32) method. Given that childhood obesity is predominantly influenced by dietary habits and physical activity levels, when children consume high-energy, particularly high-fat foods, in excess of their daily energy needs, it leads to an energy surplus and consequently, an increase in body fat accumulation, culminating in obesity (33). The high-fat diet approach to creating an obesity model more accurately reflects the dietary patterns of obese children in real-world settings, thus the study opts for the high-fat diet method.

### 4.3 Selection of observational indicators

Under typical conditions, the sexual development of female children is governed by the HPGA axis, which remains suppressed until puberty, characterized by an increase in FSH levels (34). Upon reaching puberty, the HPGA axis becomes activated, leading to an increase in LH and FSH secretion, with a predominance of LH (34). Estradiol (E2), primarily secreted by the ovaries, facilitates the proliferation of the uterine lining and the development of secondary sexual characteristics in females (35). E2 levels are initially low during early follicular development, peak pre-ovulation, then rapidly decline, and subsequently reach a second peak during the mid-luteal phase (35). As previously noted, the regulatory mechanisms of sexual development in SD rats are analogous to those in humans. Consequently, assessing levels of FSH, LH, and E2 is of considerable importance for determining the estrous cycle in rats. The vaginal opening, a hallmark of sexual maturity in female SD rats, has become a widely accepted indicator. This study continues to regard the vaginal opening as a definitive sign of sexual maturity in rats.

### 4.4 Analysis of the effectiveness of acupoint embedding

The purpose of this study was to assess the efficacy of acupoint embedding therapy in treating obesity and its potential to delay the onset of obesity-related precocious puberty. The acupoints selected for the therapy (ST44 and ST36) have been previously validated for their role in obesity treatment (36, 37). At the outset, there was no significant difference in body weight among the SD rats in the ACE, HFD, and NCD groups on the day of group assignment (postpartum day 21). However, the ACE and HFD groups, which were fed a high-fat diet, exhibited a more rapid weight gain compared to the NCD group. By the study’s conclusion (postpartum day 41), significant differences were observed among the three groups, indicating that the high-fat diet effectively simulated the dietary habits and obesity trends seen in children with unhealthy diets.

The divergence in weight change trajectories among the groups met the study’s criteria for obesity-induced precocious puberty. The ACE and HFD groups, both subjected to a high-fat diet, were expected to reach sexual maturity earlier than the NCD group. The timing of vaginal opening in all rats of the ACE and HFD groups was used as the study’s endpoint to facilitate a statistical analysis of sexual maturation timing. Kaplan-Meier survival curves (**Figure 2B**) were constructed using vaginal opening as the outcome, revealing no difference between the ACE and NCD groups but significant differences between the ACE and HFD, and HFD and NCD groups. This suggests that the ACE group’s sexual maturation was delayed relative to the HFD group.

Although vaginal opening is an indicator of sexual maturity, it is not the sole measure. In studying rat sexual development, the levels and ratios of FSH, LH, and E2 are also important indicators (38). These hormone levels and ratios exhibit specific patterns of change at different stages of sexual development (39). As shown in **Figures 2A, B**, all SD rats in the HFD group had vaginal opening by day 36 postpartum, earlier than the NCD and ACE groups, indicating precocious puberty. **Figures 3D–F** present the longitudinal changes in hormone levels in the ACE, HFD, and NCD groups. Compared to day 21 postpartum, on day 41 postpartum, the ACE group showed increased LH and E2 with no change in FSH; the HFD group showed increased LH and E2 but decreased FSH; and the NCD group showed increased LH and E2 with no change in FSH. **Figures 3A–C** present the cross-sectional comparison of hormone levels among the ACE, HFD, and NCD groups. The ACE group had lower LH, higher FSH, and higher E2 levels compared to the HFD group; similar LH, higher FSH, and similar E2 levels compared to the NCD group. The HFD group had higher LH, lower FSH, and lower E2 levels compared to the ACE group.

Based on the vaginal opening time and changes in hormone levels, it is inferred that the SD rats in the ACE group might be in late puberty to early adulthood or the pre-ovulatory phase, more likely the pre-ovulatory phase. The SD rats in the HFD group might be in the pre-ovulatory or ovulatory phase, more likely having imminent or ongoing ovulation, suggesting possible precocious puberty. The SD rats in the NCD group might be in early to mid-puberty, with some possibly entering late puberty. Taken together, the ACE group showed a sexual development process closer to the NCD group compared to the HFD group, with a later vaginal opening time, suggesting a potential delaying effect on precocious puberty.

## 5 Limitations

This experiment in SD rats showed that acupuncture point embedding might delay the onset of obesity-related precocious puberty. However, the following factors might affect the extrapolation of these results to human studies: 1) Genetic and experimental environmental factors. Experimental rats are usually selected strains with relatively consistent genetic backgrounds, whereas humans have highly diverse genetic backgrounds. The animal experiments in this study were conducted under highly controlled conditions (e.g., diet, lighting, and temperature), while humans live in diverse environments where diet, lifestyle, and social factors can affect the extrapolation of results. 2) Long-term effects of interventions. The lifespan and sexual development cycle of rats are shorter, and the long-term effects of interventions might not be entirely consistent between SD rats and humans. Therefore, when applying these study results to humans, it is recommended to conduct real-world research.

## 6 Conclusion

This study utilized a high-fat diet to simulate an SD rat model of obesity-related precocious puberty and employed acupoint embedding therapy, a treatment for obesity, to confirm its efficacy in delaying the onset of precocious puberty associated with obesity. The findings of this study offer, on one hand, a novel therapeutic approach for clinical medical staff in the prevention and treatment of obesity-related precocious puberty. On the other hand, the research also stimulates further exploration by scientific researchers into the mechanisms by which acupoint stimulation can play a role in preventing and treating precocious puberty.

## Data Availability

The raw data supporting the conclusions of this article will be made available by the authors, without undue reservation.
